# CO-Releasing Molecule-2 Prevents Acute Kidney Injury through Suppression of ROS-Fyn-ER Stress Signaling in Mouse Model

**DOI:** 10.1155/2021/9947772

**Published:** 2021-07-06

**Authors:** Md Jamal Uddin, Jeewon Jeong, Eun Seon Pak, Hunjoo Ha

**Affiliations:** Graduate School of Pharmaceutical Sciences, College of Pharmacy, Ewha Womans University, Republic of Korea

## Abstract

Acute kidney injury (AKI) most commonly appears in critically ill patients in hospitals. AKI is characterized as a quick deterioration of kidney function and has recently been identified to be tightly interlinked with chronic kidney diseases. The emerging major mediators of AKI include oxidative stress and endoplasmic reticulum (ER) stress. Carbon monoxide (CO) attenuates oxidative stress and ER stress in various cells, while Fyn, a member of the Src kinase family, is activated by oxidative stress and contributes to ER stress in skeletal muscle. Considering these, the objective of the current research was to determine (i) the involvement of Fyn in ER stress-mediated AKI and (ii) the effect of CO-releasing molecule-2 (CORM2) on reactive oxygen species- (ROS-) Fyn-ER stress-mediated AKI. Pretreatment with CORM2 (30 mg/kg) efficiently inhibited LPS (30 mg/kg)-induced oxidative stress, inflammation, and cellular apoptosis during AKI in C57BL/6J mice. Also, CORM2 efficiently suppressed the activation of Fyn and ER stress in AKI mice. Consistently, pretreatment with CORM2 inhibited oxidative stress, Fyn activation, ER stress, inflammation, and apoptosis in LPS- or H_2_O_2_-stimulated proximal epithelial tubular cells. Fyn inhibition using siRNA or an inhibitor (PP2) significantly attenuated ER stress responses in the cells. These data suggest that CORM2 may become a potential treatment option against ROS-Fyn-ER stress-mediated AKI.

## 1. Introduction

Acute kidney injury (AKI) usually appears in hospital settings. The incidence rate of AKI is increasing, and it is accountable for nearly 2 million deaths per year worldwide [1]. Collective evidence suggests that patients with a history of AKI may develop chronic kidney diseases (CKD) [2, 3]. AKI often occurs in the setting of sepsis [4]. The pathophysiology of AKI is complex and includes inflammation, tubular injury, and vascular damage [5, 6]. Also, AKI can be mediated by dysregulation of the immune system, cell death or apoptosis, mitochondrial dysfunction, oxidative stress, and endoplasmic reticulum (ER) stress [7, 8]. Importantly, increased levels of reactive oxygen species (ROS) are linked with ER stress [9, 10]. Although the pathophysiology of AKI has been studied, many targeted clinical therapies have failed [11]. Thus, urgent interventions are needed to treat AKI patients in the intensive care unit [12].

ER stress comprises a cellular process that is prompted by a variety of circumstances. ER stress is associated with the activation of three main ER stress sensors including inositol-requiring enzyme 1 (IRE1), activating transcription factor 6 (ATF6), and RNA-dependent protein kinase-like ER kinase (PERK). The activated IRE1 promotes X-box-binding protein 1 (XBP1) cleavage to make spliced XBP1 (sXBP1) [13]. The activated XBP1 stimulates inflammatory cytokine production [14]. Also, during ER stress, IRE1 promotes JNK activation [15], which leads to cellular apoptosis. Various studies have suggested that inhibition of ER stress may fight against AKI [16–18].

Src family kinases (SFKs) belong to nonreceptor protein tyrosine kinases. Yet, there are eight members of SFK including Blk, c-Src, Fgr, Fyn, Hck, Lck, Lyn, and Yes. Some of them (c-Src, Fyn, and Yes) express ubiquitously, and others (Blk, Fgr, Hck, Lck, and Lyn) are primarily found in hematopoietic cells. Among all of these, Fyn, a 59 kDa protein, has been known to regulate various cellular functions including cellular growth, survival, adhesion, motility, T-cell receptor signaling, and cytoskeletal remodeling [19–21]. The Fyn kinase has been considered a critical regulator of a variety of pathological conditions, including progressive CKD [22, 23]. Fyn has been reported to promote ER stress as well as subsequent activation of the IRE1*α*-JNK pathway, driving cell death in skeletal muscle [24]. However, the involvement of Fyn in ER stress-mediated AKI has not been studied.

Carbon monoxide (CO), an endogenous gas produced from heme degradation by heme oxygenase, shows protective functions in various pathological conditions [25–29]. CO inhibits ROS-mediated oxidative stress in human airway smooth muscle cells, human pulmonary alveolar epithelial cells, lens epithelial cells, and embryonic cells [30–34]. Also, CO/CO-releasing molecules (CORMs) suppress ER stress in endothelial cells and islet cells [35, 36]. In the mouse kidney, CO protects against obstruction-induced fibrosis [37]. Also, CO/CORMs can be advantageous for treating AKI [38, 39]. Importantly, the findings of our recent study suggested that CORM2 protects lipopolysaccharides- (LPS-) induced AKI through inhibition of ER stress in mice [16]. Yet, the detailed molecular mechanism involved in the protective effects of CO against ER stress-mediated AKI remains elusive.

Since CO attenuates oxidative stress and ER stress, and since Fyn contributes to ER stress, the aim of the current research was to verify (i) the involvement of Fyn in ER stress-mediated AKI and (ii) the effect of CORM2 on ROS-Fyn-ER stress signaling in AKI.

## 2. Materials and Methods

### 2.1. Materials

Chemicals and reagents were purchased from Sigma-Aldrich (St. Louis, MO, USA), unless otherwise stated.

### 2.2. Animals

Approval for animal studies was obtained from the institutional animal care and use committee (IACUC No. 16-055) of Ewha Womans University. Six-week-old male C57BL/6 mice (Japan SLC Inc., Hamamatsu, Japan) were divided into six groups: control, CORM2, PP2, LPS, LPS+CORM2, and LPS+PP2. Mice were pretreated with CORM2 (30 mg/kg, intraperitoneal, i.p.) or PP2 (2 mg/kg, i.p.) for 2 h and then subjected to administration of 15 mg/kg LPS (a single i.p. injection) for 18 h [16]. An equal volume of dimethyl sulfoxide (DMSO) and saline as the vehicle of CORM2/PP2 and LPS, respectively, were injected into the control mice. All mice were sacrificed at 18 h after injection of LPS via anesthesia with 16.5% urethane (10 ml/kg). After anesthesia, blood was collected in a heparin-coated syringe. After perfusion with phosphate-buffered saline (PBS), the left kidneys were washed, weighed, and then kept for further studies. The right kidneys were subjected to 2% paraformaldehyde-lysine-periodate fixation at pH 7.4 and then stored at room temperature for further study.

### 2.3. Blood Parameters

Blood plasma was obtained after centrifuging the blood samples at 900 g for 15 min at 4°C. The levels of plasma creatinine were measured using a DetectX Serum Creatinine Detection Kit (Arbor Assays, Ann Arbor, MI, USA). The plasma kidney injury molecule-1 (KIM1, MKM100, R&D Systems) and cystatin C (R&D Systems, Minneapolis, MN, USA) levels were determined using enzyme-linked immunosorbent assay (ELISA) kits. The thiobarbituric acid reaction was used to measure the levels of plasma lipid hydroperoxide (LPO) as described [40].

### 2.4. Kidney LPO

The kidney LPO was measured according to the protocols of the kit manufacturer (Cayman Chemical Co, Ann Arbor, MI, USA).

### 2.5. Histology and Immunohistochemistry

The paraffin-embedded kidney sections were then stained with a periodic acid-Schiff (PAS) reagent. Immunohistochemistry for anti-F4/80 (1 : 200; Santa Cruz Biotechnology, Inc., Santa Cruz, CA, USA), anti-nitrotyrosine (1 : 200; Santa Cruz Biotechnology), anti-NADPH oxidase 2 (NOX2, 1 : 500), and anti-NADPH oxidase 4 (NOX4, 1 : 300) antibodies was performed. A Zeiss microscope having an Axio cam HRC digital camera and software (Carl Zeiss, Thornwood, NY, USA) was utilized to take the images.

### 2.6. Immunofluorescence Staining

After deparaffinization and rehydration, tissue sections were incubated with retrieval solution and heated in a microwave to recover antigenicity. Nonspecific binding was blocked with serum-free blocking solution for 30 min at room temperature. Kidney sections were then incubated with anti-pFyn (1 : 100; Santa Cruz Biotechnology) or anti-p-Src (1 : 100; Cell Signaling Technology, Danvers, MA, USA) overnight at 4°C. Tissue sections were incubated for 1 h with Alexa 488-conjugated goat anti-mouse (1 : 1000; Invitrogen, Carlsbad, CA, USA) or Alexa 568-conjugated goat anti-rabbit (1 : 1000; Invitrogen) antibodies. Cell nuclei were detected with 4′,6-diamidino-2-phenylindole (1 : 1000; Thermo Fisher Scientific, Waltham, MA). Images were captured by a Zeiss ApoTome Axiovert 200M microscope (Carl Zeiss Microscopy GmbH, 07745, Jena, Germany).

### 2.7. Direct Measurement of ROS

To determine the presence of ROS, the frozen sections of the kidney were stained for 10 min at 37°C with 5 *μ*M dihydroethidium (DHE, red fluorescence at 561 nm, Molecular Probes, Eugene, Oregon, USA) and then stained with 4′,6-diamidino-2-phenylindole (DAPI). A Zeiss ApoTome Axiovert 200M microscope (Carl Zeiss, Germany) was used to take the images.

### 2.8. Cell Culture

Mouse proximal tubular epithelial (mProx24) cells were cultured in Dulbecco's modified Eagle's medium (DMEM) as described [41]. The cultured cells were stimulated with LPS in a time-dependent manner (0, 3, 6, 12, and 24 h). Also, the cells were treated with CORM2 (0, 5, 10, and 20 *μ*M), 10 *μ*M of PP2, 5 mM N-acetylcysteine (NAC), and 1 mM 4-phenylbutyrate (4-PBA) for 2 h and then stimulated with LPS (100 ng/ml) for 6 h or 18 h.

siRNAs were purchased from Bioneer corporation (Daejeon, South Korea). The mProx24 cells (5 × 10^5^/ml) were cultured in six-well plates for 6 h and then transfected with Src siRNA (100 nM) or Fyn siRNA (100 nM), using Lipofectamine 2000 according to the manufacturer's instructions. After transfection, the cells were starved by replacing the medium with DMEM containing 0.5% bovine serum albumin. The cells were then treated with LPS (100 ng/ml) for 6 h with or without pretreatment with CORM2 (20 *μ*M).

### 2.9. Terminal Transferase-dUTP-Nick-End Labeling (TUNEL) Assays

Apoptosis was measured using the TUNEL assay according to the manufacturer's protocol (Roche Diagnostics, Mannheim, Germany). First, after deparaffinization and rehydration, the kidney tissue was washed with PBS and then incubated with TUNEL reaction mixture for 60 min at 37°C in a humidified chamber at dark conditions. Kidney sections were subsequently washed with PBS and stained with DAPI. Images were analyzed by the Zeiss ApoTome Axiovert 200M microscope (Carl Zeiss, Germany).

In the case of mProx cells, the cultured cells in 6-well plates were treated with the mentioned reagents and then washed with 1x PBS (×2). After fixation and permeabilization, the cells were incubated with TUNEL reaction mixture for 60 min at 37°C followed by DAPI in a humidified dark chamber. Cells were subsequently washed with 1x PBS (×3) and analyzed using a Zeiss ApoTome Axiovert 200M microscope (Carl Zeiss, Germany).

### 2.10. Intracellular ROS Analysis

Intracellular ROS was measured in mProx cells according to our previous study with some modifications [42]. Briefly, the cells were washed with PBS (×3) and incubated for 30 min in the dark at 37°C PBS containing 20 *μ*M DCF-DA (Abcam, Cambridge, MA). Fluorescence of oxidized DCF was detected using a Zeiss ApoTome Axiovert 200M microscope (Carl Zeiss, Germany) at excitation wavelengths of 485 nm. The mean relative fluorescence intensity was measured by the average of five random values.

### 2.11. Real-Time RT-PCR Analysis

Real-time RT-PCR analysis for the whole kidney and mProx24 cells was performed as described previously [16]. Briefly, total RNA was isolated using the TRIzol reagent (Life Technologies, Carlsbad, CA, USA), and then, cDNA was synthesized. The mRNA expression of various genes was determined by real-time RT-PCR using an ABI7300 system (Applied Biosystems, Carlsbad, CA, USA). The primer sequences are shown in Table 1.

### 2.12. Western Blot Analysis

The protein expression was determined using Western blot analysis as described [16]. Briefly, the kidney tissue and cell lysates were separated on SDS-polyacrylamide gel electrophoresis followed by transfer to polyvinylidene difluoride membranes. Then, the membranes were incubated with various antibodies including anti-p-eIF2*α* (1 : 5000, Cell Signaling Technology), anti-p-IRE1*α* (1 : 4000, Santa Cruz Biotechnology), ATF6*α* (1 : 5000, Cell Signaling Technology), anti-pJNK (1 : 5000, Cell Signaling Technology), anti-CHOP (1 : 1000, Santa Cruz Biotechnology), anti-pSrc (1 : 1000; Cell Signaling Technology), anti-pFyn (1 : 1000; Santa Cruz Biotechnology), and anti-*β*-actin (1 : 1000) overnight at 4°C on a shaker. Then, the membranes were incubated with respective secondary antibodies, washed, and reacted with an enhanced chemiluminescent sensitive plus reaction (BioFX Laboratories, Inc., Owings Mills, MD, USA). The bands were quantified by using ImageJ software and normalized by *β*-actin.

### 2.13. Statistical Analysis

All results were expressed as the mean ± standard error (SE). The statistical differences among the groups were evaluated by one-way ANOVA and subsequent Fisher's post hoc analysis. Differences were considered to be significant when *p* < 0.05.

## 3. Results

### 3.1. CORM2 Improves Kidney Function and Attenuates Kidney Tubular Injury

**Figure 1:**
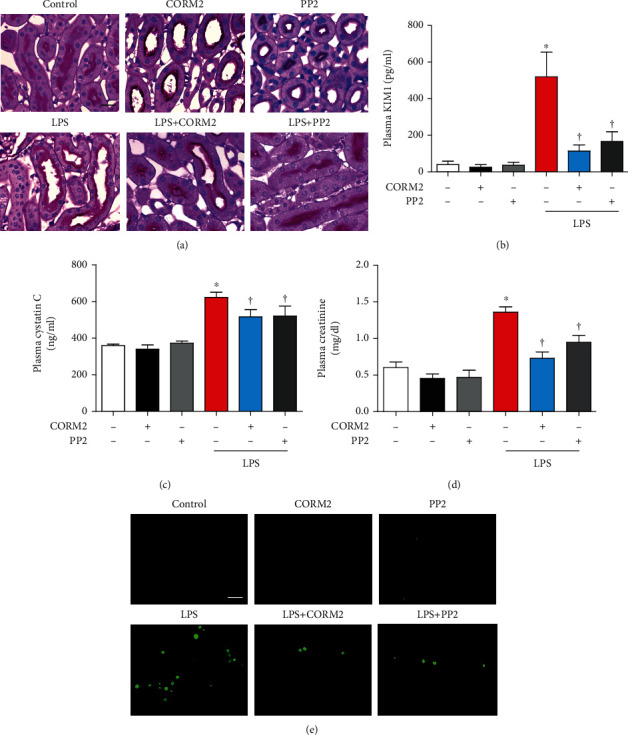
CORM2 reduces LPS-induced kidney dysfunction and injury in mice. Mice were pretreated with CORM2 (30 mg/kg) or PP2 (2 mg/kg) for 2 h and then administrated with LPS (15 mg/kg) for 18 h. (a) Paraffin-embedded kidney sections were stained with PAS (magnification: 630x; scale bar: 20 *μ*m). Blood plasma was used to determine (b) KIM1 (pg/ml), (c) cystatin C (ng/ml), and (d) creatinine (mg/dl). (e) Apoptosis was measured in paraffin-embedded kidney sections using TUNEL assays (magnification: 400x; scale bar: 100 *μ*m). Data are presented as means ± SE, control (*n* = 8), CORM2 (*n* = 8), PP2 (*n* = 8), LPS (*n* = 6-8), LPS+CORM2 (*n* = 6-8), and LPS+PP2 (*n* = 6-8); ^∗^*p* < 0.05 vs. control, ^†^*p* < 0.05 vs. LPS.

We first showed that LPS significantly decreased body weight in a time-dependent manner (Supplementary Fig. 1A). Pretreatment with CORM2 significantly inhibited the LPS-induced decrease in body weight and the kidney to body weight (Supplementary Fig. 1B-C). Then, we examined the effect of CORM2 on kidney function and morphology changes during LPS-induced AKI. The results of PAS staining (Figure 1(a)) showed tubular damage in the LPS-induced AKI mice, which was ameliorated by CORM2 or PP2. Also, CORM2 or PP2 treatment significantly inhibited the LPS-induced plasma KIM1 levels in the AKI mice (Figure 1(b)). CORM2 or PP2 significantly decreased LPS-induced plasma cystatin C (Figure 1(c)) and creatinine (Figure 1(d)) levels, suggesting that CORM2 may enhance kidney function in AKI mice. Cellular apoptosis was detected using TUNEL assays. TUNEL-positive cells were markedly increased in the LPS-induced AKI mice, and the number was decreased by CORM2 or PP2 (Figure 1(e)).

### 3.2. CORM2 Attenuates LPS-Induced Kidney Oxidative Stress and Inflammation

**Figure 2:**
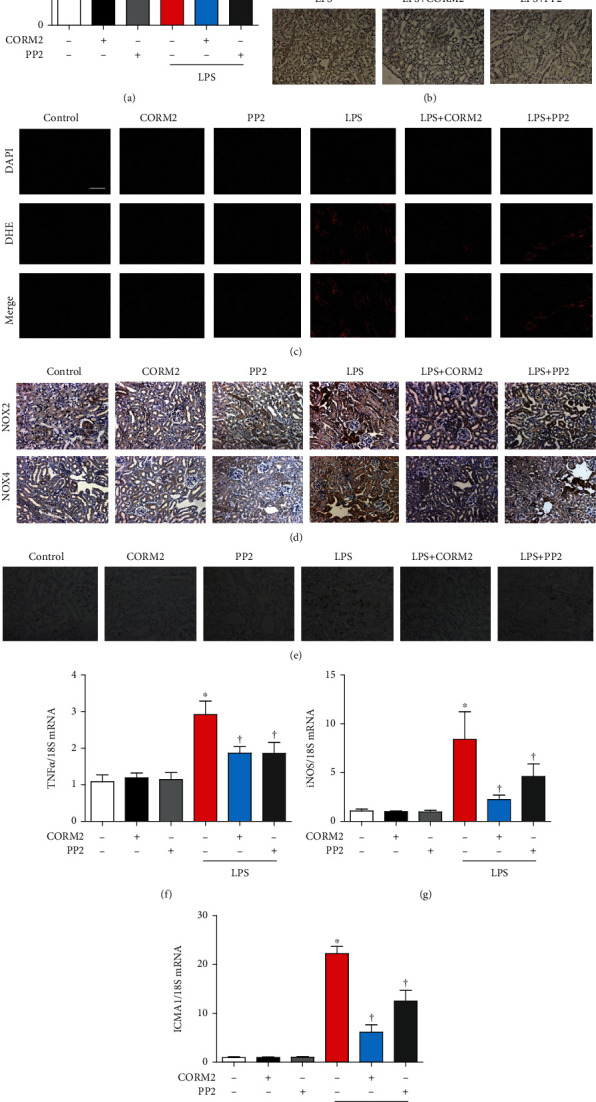
CORM2 reduces kidney oxidative stress and inflammation in LPS-induced AKI mice. (a) Blood plasma was used to determine LPO (*μ*M). (b) Anti-nitrotyrosine antibody (1 : 200) was used to stain the paraffin sections of the kidney, and accumulation of nitrotyrosine is shown in the cytosol (brown color). (c) DHE (5 *μ*M for 10 min) was used to stain the frozen sections of the kidney, and the red color indicates ROS accumulation. Magnification: 100x; scale bar: 50 *μ*m. (d, e) Paraffin sections of the kidney were incubated with (d) anti-NOX2 (1 : 500) and anti-NOX4 (1 : 400) and (e) anti-F4/80 (1 : 200) antibodies. Magnification of all images from paraffin sections: 100x; scale bar: 100 *μ*m. (f-h) Real-time RT-PCR was used to measure the mRNA levels of TNF*α*, iNOS, and ICAM1 in the kidney. Data are presented as means ± SE, control (*n* = 8), CORM2 (*n* = 8), PP2 (*n* = 8), LPS (*n* = 6-8), LPS+CORM2 (*n* = 7-8), and LPS+PP2 (*n* = 6-8); ^∗^*p* < 0.05 vs. control, ^†^*p* < 0.05 vs. LPS.

Oxidative stress is an important contributor to the pathogenesis of AKI [43]. First, we have confirmed the time-dependent effects of LPS on antioxidants. Expression of NRF2 and NQO1 mRNA was effectively decreased at 12 and 24 h after injection of LPS (Supplementary Fig. 3C). Plasma LPO was significantly increased in LPS-induced AKI mice, which was effectively inhibited by CORM2 or PP2 (Figure 2(a)). LPS-induced accumulation of nitrotyrosine in the kidney was also considerably inhibited by CORM2 or PP2 (Figure 2(b)).

To precisely measure ROS in kidney tissues, DHE staining was accomplished. LPS effectively enhanced ROS levels, which were significantly reduced by CORM2 or PP2 (Figure 2(c)). The expression levels of NOX2 and NOX4 as measured by immunostaining were significantly increased in LPS-induced AKI mice. Interestingly, these changes were significantly hindered by CORM2 or PP2 (Figure 2(d)). Also, the NOX2 and NOX4 mRNA levels were significantly decreased by CORM2 in AKI mice (Supplementary Fig. 3E-F). Together, these data suggest that CORM2 decreases oxidative stress as much as PP2 does in LPS-induced AKI.

Since inflammation is critically involved in AKI, we determined whether CORM2 or PP2 has an anti-inflammatory effect on LPS-induced AKI. First, we confirmed the time-dependent effects of LPS on proinflammatory genes. As expected, proinflammatory genes were significantly increased at various time points by both agents in the mice (Supplementary Fig. 3A). LPS effectively increased the staining of F4/80, a marker of macrophage infiltration, which was significantly reduced by treatment of CORM2 or PP2 in the kidneys (Figure 2(e)). Likely, CORM2 significantly reduced LPS-induced mRNAs of proinflammatory cytokines including TNF*α*, iNOS, and ICAM1 in the kidneys (Figure 2(f)–2(h)).

### 3.3. CORM2 Inhibits LPS-Induced Kidney ER Stress

**Figure 3:**
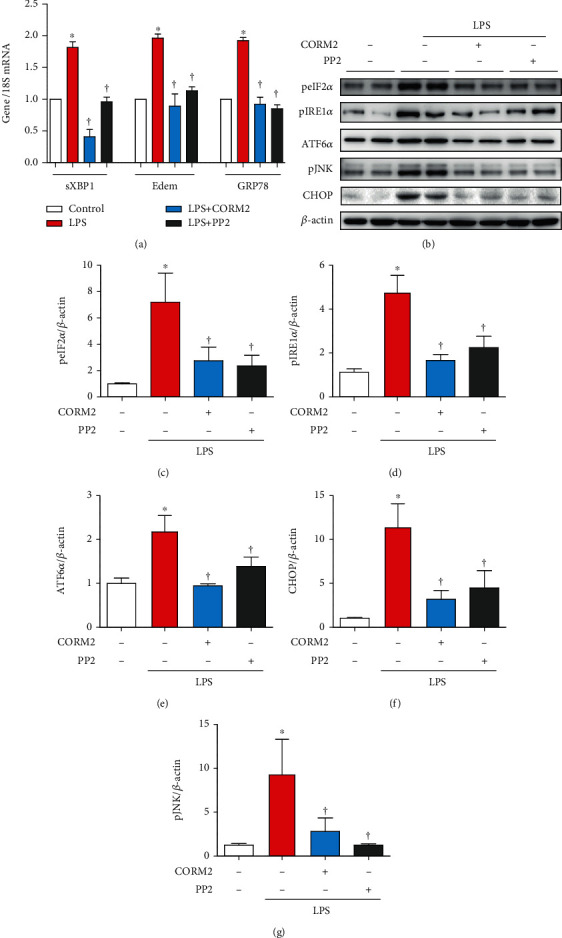
CORM2 inhibits kidney ER stress in LPS-induced AKI mice. (a) The mRNA levels of sXBP1, ER-degradation-enhancing alpha-mannosidase-like protein-1 (Edem1), and 78 kDa glucose-regulated protein (GRP78) were assessed in the kidney using real-time RT-PCR. (b) Western blotting was employed to determine the protein expression of peIF2*α*, pIRE1*α*, ATF6*α*, pJNK, and CHOP in the kidney, and (c–g) ImageJ software was used to determine the band intensities. Data are presented as means ± SE, control (*n* = 8), LPS (*n* = 7-8), LPS+CORM2 (*n* = 8), and LPS+PP2 (*n* = 8); ^∗^*p* < 0.05 vs. control and ^†^*p* < 0.05 vs. LPS.

ER stress has been reported as a hallmark of AKI [17, 18]. Thus, we have investigated the effect of CORM2 on ER stress. LPS treatment significantly increased the mRNA levels of ER stress markers including sXBP1, Edem1, and GRP78 in the kidney of AKI mice (Figure 3(a)). These were all considerably reduced by CORM2 or PP2 (Figure 3(a)). Consistently, the protein expression levels of peIF2*α*, pIRE1*α*, ATF6*α*, pJNK, and CHOP were significantly increased in AKI mice (Figures 3(b)–3(g)). As expected, these changes were significantly suppressed by CORM2 or PP2 (Figures 3(b)–3(g)).

### 3.4. CORM2 Inhibits LPS-Induced SFK Activation

**Figure 4:**
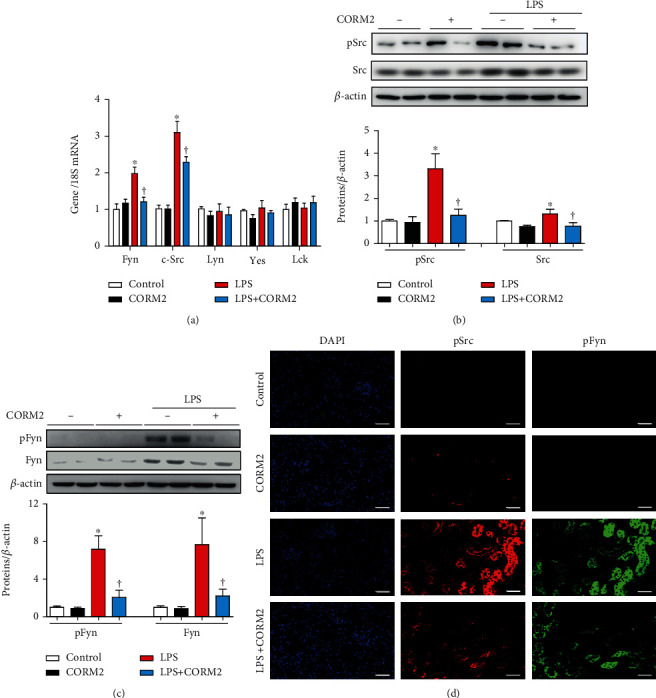
CORM2 inhibits SFK activation in LPS-induced AKI mice. (a) The mRNA levels of SFKs including Fyn, c-Src, Lyn, Yes, and Lck were determined in the kidney using real-time RT-PCR. (b, c) Western blotting analysis of phosphorylated or total Src and Fyn kinase in the kidney was performed. ImageJ software was used to detect the band intensities, and the levels of the proteins were normalized to *β*-actin. (d) Paraffin sections of the kidney were incubated with phospho-Fyn (red) and phospho-Src (green) antibodies and 4′,6-diamidino-2-phenylindole (DAPI; blue). Magnification: 100x; scale bar: 50 *μ*m. Data in the graph are presented as means ± SE, control (*n* = 8), CORM2 (*n* = 8), LPS (*n* = 7-8), and LPS+CORM2 (*n* = 7-8); ^∗^*p* < 0.05 vs. control, ^†^*p* < 0.05 vs. LPS.

As shown in Figure 4(a), the mRNA levels of SFKs such as Fyn and c-Src were significantly increased by LPS while CORM2 effectively inhibited these effects. The protein expression levels of pFyn, Fyn, pSrc, and Src were markedly increased by LPS treatment (Figures 4(b) and 4(c)) and were decreased by pretreatment with CORM2 in AKI mice (Figures 4(b) and 4(c)). In addition, immunostaining of pSrc and pFyn was markedly detectable in the kidney of LPS-induced AKI mice (Figure 4(d)), and their expression levels were decreased by CORM2 treatment (Figure 4(d)). These data show that CORM2 inhibits not only pFyn and p-c-Src but also total Fyn and c-Src in the kidney of LPS-induced AKI mice.

### 3.5. CORM2 Reduces LPS-Induced ER Stress through Inhibition of Fyn

CO attenuates ER stress [35, 36], while Fyn contributes to ER stress [24]. Since the importance of tubular epithelial cells in AKI pathophysiology is established [44], the mProx cells have been utilized to validate the protective mechanisms involved in CORM2. We have, first, confirmed the cytotoxic effects of LPS on mProx cells using MTT assays. There was no significant cytotoxic effect up to 1 *μ*g/ml LPS (Supplementary Fig. 2A). Also, there were no significant cytotoxic effects of CORM2 at different concentrations in LPS-treated cells (Supplementary Fig. 2B). Based on these observations, we have chosen the doses of CORM2 (20 *μ*M) and LPS (100 nM) for the studies with mProx cells.

**Figure 5:**
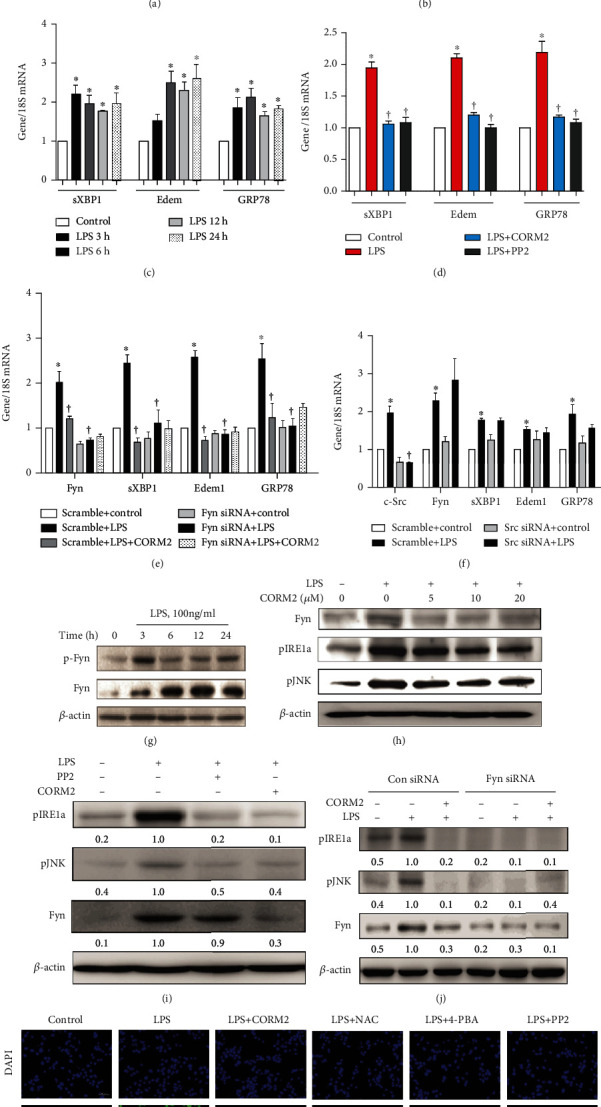
CORM2 inhibits ER stress through suppression of Fyn in LPS-treated mProx cells. (a, c) Cells were stimulated with LPS (100 ng/ml) in a time-dependent manner (0, 3, 6, 12, and 24 h). (a) SFK such as Fyn, c-Src, Lyn, Yes, and Lck mRNA levels were measured in the cells. (b, h) Cells were pretreated with CORM2 in a concentration-dependent manner (0, 5, 10, and 20 *μ*M) for 2 h and then incubated with LPS (100 ng/ml) for 6 h or 18 h. (b) The mRNA levels of Fyn and c-Src were measured at 6 h. (c) mRNAs of sXBP1, Edem, and GRP78 were measured. (d, i) Cells were pretreated with CORM2 (20 *μ*M) or PP2 (10 *μ*M) for 2 h and then stimulated with LPS (100 ng/ml) for 6 h or 24 h. (d) mRNAs of sXBP1, Edem, and GRP78 were measured. (e, f, j) Cells were transfected with control siRNA and Fyn or c-Src siRNA. Then, they were pretreated with or without CORM2 and incubated with LPS (100 ng/ml) for 6 h or 18 h. (e) Fyn siRNA (LPS, 6 h): mRNAs of Fyn, sXBP1, Edem, and GRP78 were measured. (f) c-Src siRNA (LPS, 6 h): mRNAs of c-Src, Fyn, sXBP1, Edem, and GRP78 were measured. (g) Cells were treated with LPS (100 ng/ml) in a time-dependent manner (0, 3, 6, 12, and 24 h), and protein expression of pFyn and Fyn was measured. (h) Protein expression of Fyn, pIRE1*α*, and pJNK at 18 h. (i) Protein expression of Fyn, pIRE1*α*, and pJNK at 18 h. (j) Fyn siRNA (LPS, 18 h): protein expression of Fyn, pIRE1*α*, and pJNK. (k) Cells were pretreated with CORM2 (20 *μ*M) or NAC (5 mM) or 4-PBA (1 mM) or PP2 (10 *μ*M) for 2 h and then incubated with LPS (100 ng/ml) 18 h followed by TUNEL assays. Magnification: 200x; scale bar: 50 *μ*m. All mRNAs were analyzed using real-time RT-PCR, and the proteins were measured using Western blotting analysis. ImageJ software was utilized to detect the band intensities, and the levels of the proteins were normalized to *β*-actin. Representative protein bands are shown. Data are presented as the mean ± SE, *n* = 4; ^∗^*p* < 0.05 vs. control, ^†^*p* < 0.05 vs. LPS.

Time-dependent treatment with LPS increased the mRNA expression levels of SFK (such as Fyn and c-Src) at 3 and 6 h (Figure 5(a)), and also, the mRNAs of ER stress-responsive genes (such as sXBP1, Edem, and GRP78) were significantly increased at 6, 12, and 24 h (Figure 5(c)) in mProx cells. Also, time-dependent treatment with LPS significantly increased the mRNAs of proinflammatory genes such a TNF*α*, MCP1, iNOS, and ICAM1 (Supplementary Fig. 3B) and decreased antioxidant enzymes including NRF2 and NQO1 (Supplementary Fig. 3D) in mProx cells. Pretreatment with CORM2 at different doses (0, 5, 10, and 20 *μ*M) attenuated LPS-induced upregulation of the mRNAs of c-Src and Fyn (Figure 5(b)). LPS increased pFyn and Fyn protein expression at different times (Figure 5(g)). Furthermore, pretreatment with CORM2 at different doses (0, 5, 10, and 20 *μ*M) attenuated LPS-induced upregulation of the protein expression of Fyn, pIRE1*α*, and pJNK (Figure 5(h)).

Pretreatment with CORM2 or PP2 for 2 h attenuated LPS-induced upregulation of the mRNA expression of sXBP1, Edem, GRP78 (Figure 5(d)), and CHOP (Supplementary Fig. 4A) as well as the expression levels of Fyn, pIRE1*α*, and pJNK proteins (Figure 5(i)) in mProx cells. As expected, pretreatment with iCORM2 did not inhibit LPS-induced ER stress (Supplementary Fig. 4B-C), supporting the effect of CO on ER stress. LPS significantly increased the mRNA expression levels of Fyn and c-Src in the respective control siRNA groups, and this effect was significantly decreased by CORM2 (Figures 5(e) and 5(f)). However, LPS failed to increase Fyn and c-Src expression under Fyn siRNA (Figure 5(e)) and c-Src siRNA (Figure 5(f)), respectively, indicating the specificity of transfection in the mProx cells. Importantly, LPS significantly increased the mRNA expression levels of sXBP1, Edem, and GRP78 in the respective control siRNAs, effects that were significantly suppressed by CORM2 (Figures 5(e) and 5(f)). Interestingly, LPS failed to increase the mRNA expression levels of sXBP1, Edem, and GRP78 under Fyn siRNA (Figure 5(e)), but not c-Src siRNA (Figure 5(f)). In addition, LPS failed to increase the protein expression of ER stress response proteins such as pIRE1*α* and pJNK under Fyn siRNA (Figure 5(j)), indicating the potential role of Fyn in the ER stress response in AKI. To verify the influence of CORM2 on ER stress-mediated cell death, we performed TUNEL assays. LPS significantly increased the number of TUNEL-positive mProx cells, an effect that was effectively inhibited by CORM2, NAC, 4-PBA, and PP2 (Figure 5(k)).

### 3.6. CORM2 Decreases LPS-Induced Fyn Activation through Inhibition of ROS

Fyn is induced by ROS through activation of NADPH oxidase [45], while CO is known to inhibit ROS by conformational changes of NADPH oxidase [30–32]. In addition, CO inhibits H_2_O_2_-induced oxidative stress [33]. CORM2 or PP2 significantly reduced LPS-induced plasma LPO (Figure 2(a)), nitrotyrosine accumulation (Figure 2(b)), ROS levels (Figure 2(c)), and NOX2 and NOX4 expression (Figure 2(d)) in AKI mice. This evidence and our results suggest that CORM2 may suppress Fyn through inhibition of ROS.

We have checked the cytotoxic effect of H_2_O_2_ on mProx cells using MTT assays. There was no significant cytotoxic effect of H_2_O_2_ up to 200 *μ*M on mProx cells at the 6 and 24 h time points (Supplementary Fig. 5A-B). H_2_O_2_ at 400 *μ*M showed a cytotoxic effect at 24 h but not at 6 h. Also, H_2_O_2_ significantly increased the mRNA expression levels of Fyn, c-Src, Edem, and GRP78 in mProx cells in a time- and dose-dependent manner (Supplementary Fig. 5C-D). Based on these results, we decided on the time and dose for H_2_O_2_ treatment of the cells.

**Figure 6:**
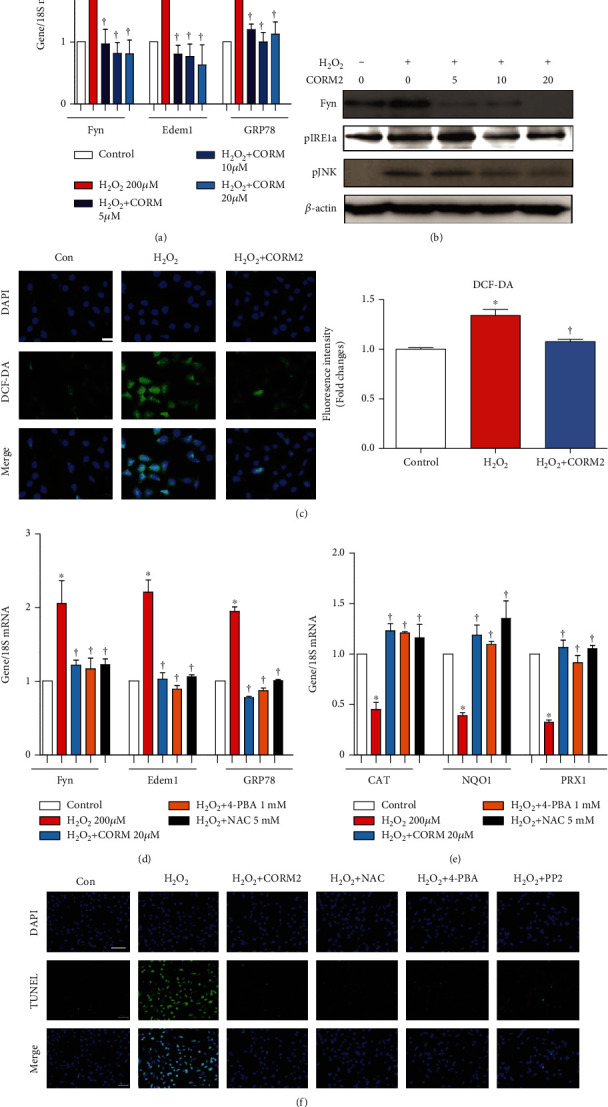
CORM2 inhibits Fyn activation through suppression of ROS in H_2_O_2_-treated mProx cells. (a, b) Cells were pretreated with CORM2 in a concentration-dependent manner (0, 5, 10, and 20 *μ*M) for 2 h and then stimulated with H_2_O_2_ (200 *μ*M) for 6 h or 18 h. (a) mRNAs of Fyn, Edem, and GRP78 were measured at 6 h. (b) Proteins of Fyn, pIRE1*α*, and pJNK were measured at 18 h. (c) The cells were pretreated with 20 *μ*M CORM2 for 2 h and then stimulated with 200 *μ*M H_2_O_2_ for 1 h. Then, cells were incubated with DCF-DA for 30 min for measuring ROS within the cells. Fold changes of fluorescence intensity were measured from five random values from 3 independent experiments. (d–f) Cells were pretreated with CORM2 (20 *μ*M), NAC (5 mM), 4-PBA (1 mM), or PP2 (10 *μ*M) for 2 h and then stimulated with H_2_O_2_ (200 *μ*M) for 6 h or 18 h. (d) mRNAs of Fyn, Edem, and GRP78 were measured at 6 h. (e) mRNAs of CAT, NQO1, and PRX1 were measured at 6 h. (f) TUNEL assay at 18 h. Magnification: 200x; scale bar: 50 *μ*m. All mRNAs were measured using real-time RT-PCR, and the proteins were measured using Western blotting analysis. Representative protein bands are shown. Data are presented as the mean ± SE, *n* = 4; ^∗^*p* < 0.05 vs. control, ^†^*p* < 0.05 vs. H_2_O_2_.

To confirm the role of ROS on Fyn, we pretreated the mProx cells with CORM2 at different doses (0, 5, 10, and 20 *μ*M). Various doses of CORM2 significantly reduced the H_2_O_2_-induced mRNA expression levels of Fyn, Edem, and GRP78 (Figure 6(a)) and the protein expression levels of Fyn, pIRE1*α*, and pJNK (Figure 6(b)). Also, H_2_O_2_-induced pFyn expression was inhibited by CORM2 at 1 h (Supplementary Fig. 5E). Interestingly, CORM2 effectively inhibited H_2_O_2_-induced ROS production in the cells (Figure 6(c)). Pretreatment with CORM2, 4-phenylbutyric acid (4-PBA, an ER stress inhibitor), or N-acetylcysteine (NAC, an antioxidant) significantly decreased the H_2_O_2_-induced upregulation of the mRNA expression levels of Fyn, Edem, and GRP78 (Figure 6(d)). Also, pretreatment with CORM2, 4-PBA, or NAC significantly decreased the H_2_O_2_-induced upregulation of the mRNA expression of catalase (CAT), NQO1, and PRX1 (Figure 6(e)). Consistently, H_2_O_2_ significantly increased the number of TUNEL-positive mProx cells, and this was effectively inhibited by CORM2, NAC, 4-PBA, and PP2 (Figure 6(f)).

## 4. Discussion

The current data demonstrate that the treatment of CORM2 exerts a protective effect against LPS-induced AKI by reducing ROS-Fyn-mediated ER stress.

Since the LPS model mimics various aspects of sepsis in humans [46], we have used LPS-induced AKI as an AKI mouse model instead of some other models [38, 47–51]. The protective effects of CORM2 on LPS-induced AKI are coherent with earlier findings [38, 47–51]. CO/CORMs protect against AKI by inhibiting inflammation, oxidative injury, and cellular apoptosis [38, 47–51]. Considering the protective effects of CORM2 on AKI, we further dissected the mechanisms, focusing on Fyn-ER stress.

CO reduces ER stress in diverse cell types [35, 36]. Considering that ER stress is associated with kidney tubular epithelial cell apoptosis and injury [7] and that LPS mediates ER stress leading to AKI [43], we have measured peIF2*α*, pIRE1*α*, ATF6*α*, pJNK, and CHOP expression in kidney tissues. Under our experimental conditions, LPS-induced expression of peIF2*α*, pIRE1*α*, ATF6*α*, pJNK, and CHOP was efficiently hindered by CORM2, suggesting a protective effect of CORM2 on ER stress-associated AKI.

Although Fyn is known to be an important regulator in cancer biology [52], recent studies have indicated the involvement of SFKs, including Fyn, in the pathogenesis of progressive CKD [22, 23]. Genetic or pharmacological inhibition of Fyn attenuates kidney fibrosis by inhibition of phospho-STAT3 in UUO mice [23]. Fyn overexpression activates mammalian target of rapamycin complex 1 (mTORC1) through inhibition of the LKB1-AMPK pathway, leading to skeletal muscle atrophy [53]. Fyn promotes ER stress and consecutive activation of the IRE1*α*-JNK pathway, driving cell death in skeletal muscle [24]. Fyn activates mTORC1 [53] and IRE1*α*-JNK [24] pathways. While mTOR is associated with ER stress in kidney tubular cells [55], Fyn has been suggested as a potential therapeutic target in AKI [54]. Thus, it is suggested that suppression of Fyn may attenuate AKI through inhibition of ER stress. Accordingly, the present study showed that pharmacologic or genetic inhibition of Fyn suppressed ER stress responses in the kidney of LPS-treated AKI mice as well as in mProx cells.

ROS is associated with ER stress [9, 10]. The inhibition of ROS by NAC effectively attenuated ER stress responses in IRI-induced AKI in mice [56]. NADPH oxidase (NOX) enzyme complexes are endogenous sources of ROS such as O_2_^−^ and H_2_O_2_. Inhibition of NOX1 leads to attenuation of elevated ROS levels in *in vitro* models of atopic dermatitis (AD) and psoriasis (PSO) in keratinocytes [57]. CO is known to inhibit ROS by conformational changes in NOX [30–32]. CO-releasing molecules such as CORM2 and CORM-401 effectively inhibit H_2_O_2_-induced ROS production in the murine intestinal epithelial MODE-K cells [58]. Consistent with this, CORM2 significantly reduced H_2_O_2_-induced ROS production in mProx cells under our experimental conditions. Yet, CORM3 had no direct inhibitory effects on the H_2_O_2_ concentration in a test tube [33]. Since generation of ROS is linked with ER stress, CO may perform a vital role in the reduction in ROS-mediated ER stress during AKI. In the current study, CORM2 treatment not only efficiently reduced oxidative stress but also significantly enhanced antioxidant gene levels in LPS-treated AKI mice. Under oxidative stress conditions, ROS increases SKFs, including Fyn expression [45]. ROS (such as H_2_O_2_) directly oxidizes cysteine 488 of Fyn resulting in increased Fyn kinase activity in human epidermal keratinocytes and murine embryonic fibroblasts and subsequently activates JNK, a downstream target of Fyn [59]. Consistently, in our study, LPS or H_2_O_2_ effectively increased pJNK and other ER stress markers, as well as cellular apoptosis. Interestingly, these effects were all inhibited by CORM2, NAC, 4-PBA, and PP2. CORM2 also suppressed LPS- or H_2_O_2_-induced Fyn and c-Src activation. In addition, LPS failed to increase ER stress responses under Fyn siRNA, but not c-Src siRNA. These data indicate the potential suppressive effect of CO on the ROS-Fyn-ER stress axis in AKI.

However, there are several unanswered questions such as the following: (a) the delayed treatment of CORM2 on AKI was not studied, (b) the detailed mechanisms involved in the inhibition of H_2_O_2_-mediated Fyn-ER stress signaling by CORM2 have not been explored, and (c) protocol of optimal delivery and dosing and the possible treatment of CO in human diseases have not been recognized yet. Further, toll-like receptors (TLRs), mainly TLR4, are an important player in inflammation and many other cellular processes in the progression of AKI [60]. Treatment of CORM2 attenuated the levels of TLR2 and TLR4/ROS and reduced inflammation in the lung [61]. Thus, it needs to examine whether CORM2 can attenuate AKI via inhibition of TLRs. In addition, premature senescence estimated by *β*-galactosidase and p53/p21 expression in NRK52E cells [62] and M1 macrophage polarization in RAW264.7 macrophages [63] have recently showed to play a role in AKI. Therefore, the effect of CORM2 on LPS-induced senescence and macrophage polarization in AKI needs to be investigated.

**Figure 7:**
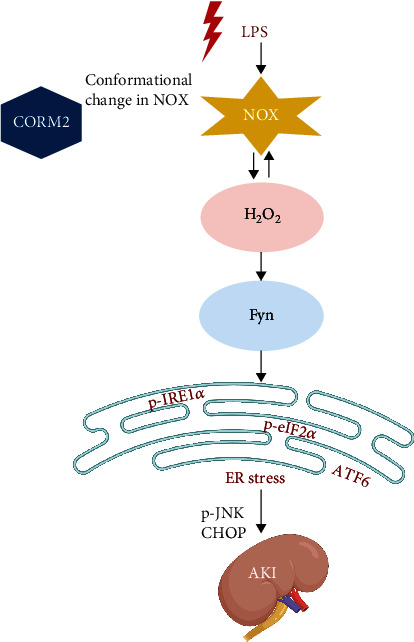
Suggested schematic diagram for the effect of CO on ROS-Fyn-ER stress activation in LPS-induced AKI. LPS mediates ROS leading to Fyn-ER stress signaling, which activates apoptosis signaling (such as CHOP and pJNK) in AKI. CO attenuates ER stress through suppression of Fyn activation in AKI. LPS-induced ROS is connected to activation of Fyn, while CO is known to reduce ROS levels. Thus, CO may become an alternative treatment option against Fyn-mediated ER stress in AKI.

In conclusion, our results and existing evidence supports that stress stimuli (such as LPS)-induced oxidative stress mediates Fyn-ER stress signaling and that inhibition of oxidative stress by CORM2 may have a protective effect against AKI (Figure 7). In the present study, LPS significantly induced kidney injury, oxidative stress, ER stress, tubular apoptosis, and inflammation, which were all inhibited by CORM2 in AKI mice and mProx cells. In addition, CORM2 suppressed LPS- or H_2_O_2_-induced Fyn activities in vivo and in vitro. LPS failed to increase ER stress responses under Fyn siRNA, but not c-Src siRNA, indicating the potential role of Fyn in the ER stress response in AKI. These findings suggest that treatment of CORM2 aimed at preventing ROS-mediated Fyn-ER stress signaling may become a promising option to treat AKI.

## Figures and Tables

**Table 1:** Primers used for real-time RT-PCR analysis.

Gene (mouse)	Primer sequences
CAT	F 5′-CACACCTACACGCAGGCCGG-3′ R 5′-CTGCGCTCCGGAGTGGGAGA-3′
Edem1	F 5′-TGGGTTGGAAAGCAGAGTGGC-3′ R 5′-TCCATTCCTACATGGAGGTAGAAGGG-3′
Fyn	F 5′-CTTTGGGGGTGTGAACTCCT-3′ R 5′-TTCTGCCTGGATGGAGTCAA-3′
Hck	F 5′-AGGGGTTAGGACTGGGAACA-3′ R 5′-CCCCAGAGATTTTGGACCCC-3′
iNOS	F 5′-ATGTCCGAAGCAAACATCAC-3′ R 5′-TAATGTCCA GGAAGTAGG TG-3′
ICAM1	F 5′-TGCCTCGGGAATGGAAAG-3′ R 5′-ATGGTAGTCTCCCCATCGTCATA-3′
GRP78	F 5′-AGCCATTGGATCACAACCTC-3′ R 5′-AGAAGCGAGAGATCCATCCA-3′
Lck	F 5′-ACGATCTCGGGGATCATGG-3′ R 5′-GAGATCTTGCTGTCCAGTGGG-3′
Lyn	F 5′-AGCTCCAGAGGCCATCAACT-3′ R 5′-CACATCTGCGTTGGTTCTCC-3′
c-Src	F 5′-TCCACACCTCTCCGAAGCAA-3′ R 5′-CATGCTGATGGCCTGTGTCA-3′
NQO1	F 5′-TTCTCTGGCCGATTCAGAG-3′ R 5′-GGCTGCTTGGAGCAAAATAG-3′
Prx1	F 5′-TGGCCAACGAAGGGGTTAAA-3′ R 5′-GATGAGGCTGCAGTTGAGGT-3′
sXBP1	F 5′-GAGTCCGCAGCAGGTG-3′ R 5′-GTGTCAGAGTCCATGGGA-3′
TNF*α*	F 5′-CGTCAGCCGATTTGCTATCT-3′ R 5′-CGGACTCCGCAAAGTCTAAG-3′
18S	F 5′-CGAAAGCATTTGCCAAGAAT-3′ R 5′-AGTCGGCATCGTTTATGGTC-3′

F: forward; R: reverse.

## Data Availability

All data could be found within the manuscript.

## Supplementary Materials

Supplementary Materials Supplementary Fig. 1: CORM2 improves body weight and kidney to body weight in AKI mice. Supplementary Fig. 2: effect of LPS and CORM2 on the viability of mProx cells. Supplementary Fig. 3: effect of LPS or CORM2 on inflammation and oxidative stress in mice and mProx cells. Supplementary Fig. 4: effect of CORM2 on ER stress signaling in mProx cells. Supplementary Fig. 5: effect of H_2_O_2_ on cell viability and ER stress responses in mProx cells.
