# Supplementary material for: CO-Releasing Molecule-2 Prevents Acute Kidney Injury through Suppression of ROS-Fyn-ER Stress Signaling in Mouse Model
Source: Oxid Med Cell Longev. 2021 Jul 6;2021:9947772. doi: 10.1155/2021/9947772 (PMC8277502; doi:10.1155/2021/9947772)
Supplement: Supplementary Materials — Supplementary Fig. 1: CORM2 improves body weight and kidney to body weight in AKI mice. Supplementary Fig. 2: effect of LPS and CORM2 on the viability of mProx cells. Supplementary Fig. 3: effect of LPS or CORM2 on inflammation and oxidative stress in mice and mProx cells. Supplementary Fig. 4: effect of CORM2 on ER stress signaling in mProx cells. Supplementary Fig. 5: effect of H2O2 on cell viability and ER stress responses in mProx cells. [file 9947772.f1.docx]

**SUPPLEMENTARY MATERIALS**

**Supplementary methods**

*Animals*

Six-week-old male C57BL/6 mice (Japan SLC Inc., Hamamatsu, Japan) were divided into four groups: (i) control, (ii) LPS 6 h, (iii) LPS 12 h, and (iv) LPS 24 h. Induction of AKI was evaluated at 6, 12, and 24 h after a single intraperitoneal (i.p.) injection of LPS (15 mg/kg).

*MTT assay*

Cell viability was measured using MTT (3-[4, 5-dimethylthiazol-2-yl]-2, 5-diphenyl tetrazolium bromide) assays. After reaching 70% confluence, the mProx cells in 96-well plates were treated with LPS or H_2_O_2_ at the indicated times and concentrations. And then, the medium was replaced with medium containing 1 mg/ml of MTT (Sigma) according to the manufacturer's instructions. To quantify the cell viability, MTT formazan was eluted with isopropanol and the absorbance of the samples was detected at 570 nm, using 690 nm as a reference wavelength, with a spectrophotometric ELISA plate reader.

**Supplementary figure and legends**


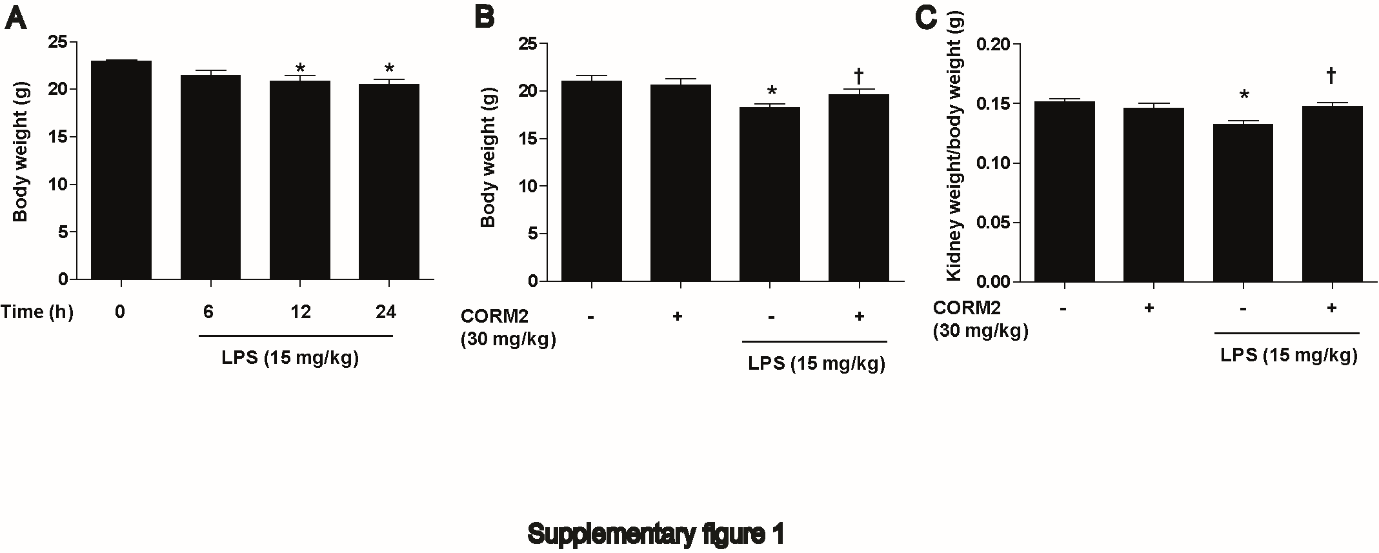
**Supplementary Fig. 1.** CORM2 improves body weight and kidney to body weight in AKI mice. (A) Mice were treated with LPS (15 mg/kg) in a time-dependent manner (0, 6, 12, and 24 h) and their body weight was measured (g). (B-C) Mice were pretreated with CORM2 (30 mg/kg) or PP2 (2 mg/kg) for 2 h and then treated with LPS (15 mg/kg) for 18 h. (B) Body weight (g), and (C) kidney to body weight (g). Data are presented as means ± SE of 6–8 mice/group; *p < 0.05 vs. control, †p < 0.05 vs. LPS.


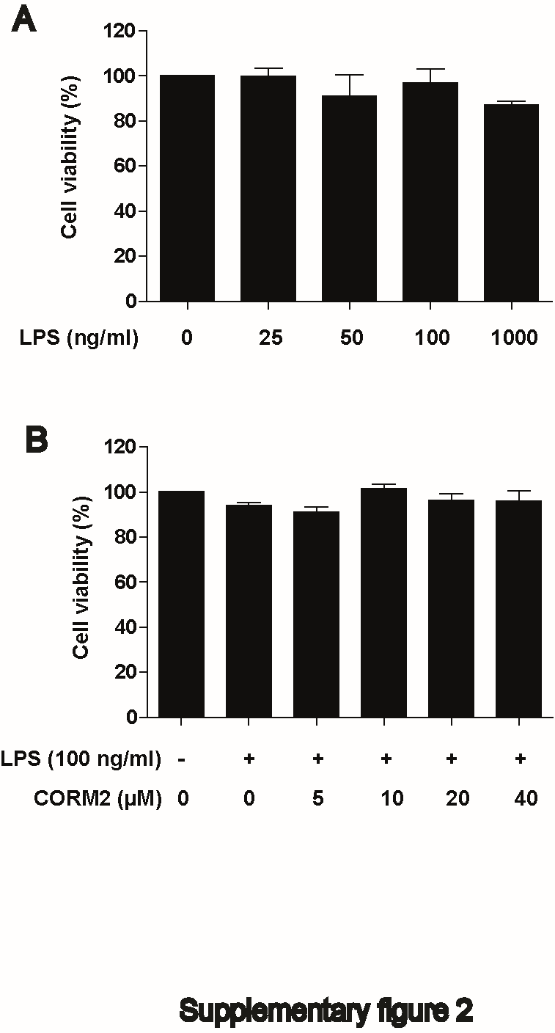


**Supplementary Fig. 2.** Effect of LPS and CORM2 on the viability of mProx cells. (A) Cells were stimulated with LPS in a dose-dependent manner (0, 25, 50, 100, and 1000 ng/ml) for 24 h and viability (%) was checked by MTT assays. (B) Cells were pretreated with CORM2 in a concentration-dependent manner (0, 5, 10, 20, and 40 µM) for 2 h and then stimulated with LPS (100 ng/ml) for 24 h. Cell viability (%) was checked by MTT assays. Data are presented as mean ± SE, n=4. *p<0.05 vs. control.


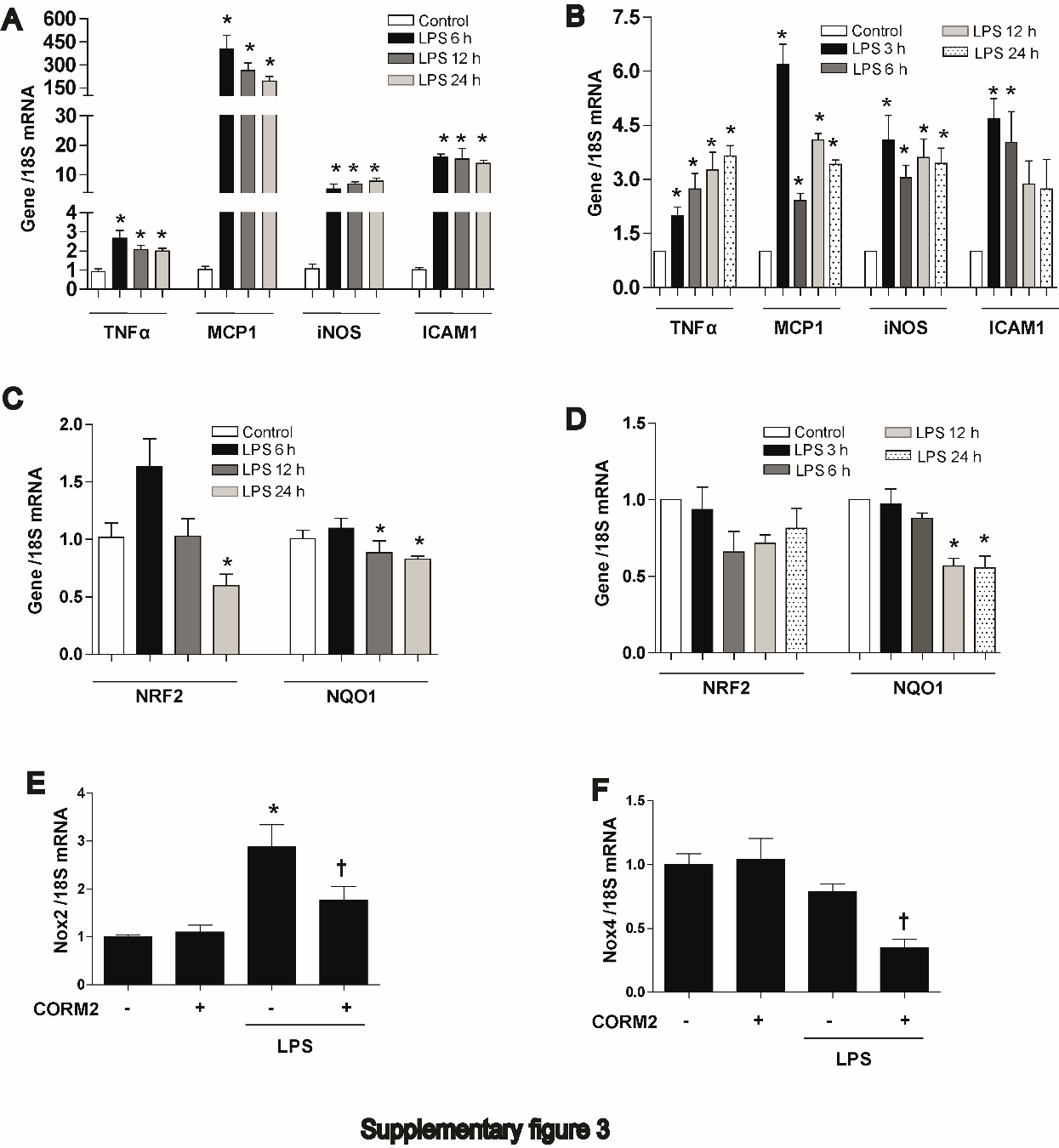


**Supplementary Fig. 3.** Effect of LPS or CORM2 on inflammation and oxidative stress in mice and mProx cells. (A, C) Mice were administered LPS (15 mg/kg) in a time-dependent manner (0, 6, 12, and 24 h). (A) mRNAs of TNFα, MCP1, iNOS, and ICAM1. (C) mRNAs of NRF2 and NQO1. (B, D) Cells were stimulated with LPS (100 ng/ml) in a time-dependent manner (0, 3, 6, 12, and 24 h). (B) mRNAs of TNFα, MCP1, iNOS, and ICAM1. (D) mRNAs of NRF2 and NQO1. (E-F) Mice were pretreated with CORM2 (30 mg/kg) for 2 h and then stimulated with LPS (15 mg/kg) for 18 h. (E) Nox2 mRNA, and (F) Nox4 mRNA. All mRNAs were measured using real-time PCR. Data are presented as means ± SE of 6–8 mice/group; *p < 0.05 vs. control, †p < 0.05 vs. LPS (mouse). In addition, data are presented as mean ± SE, n=4. *p<0.05 vs. control, †p < 0.05 vs. LPS (mProx cells).


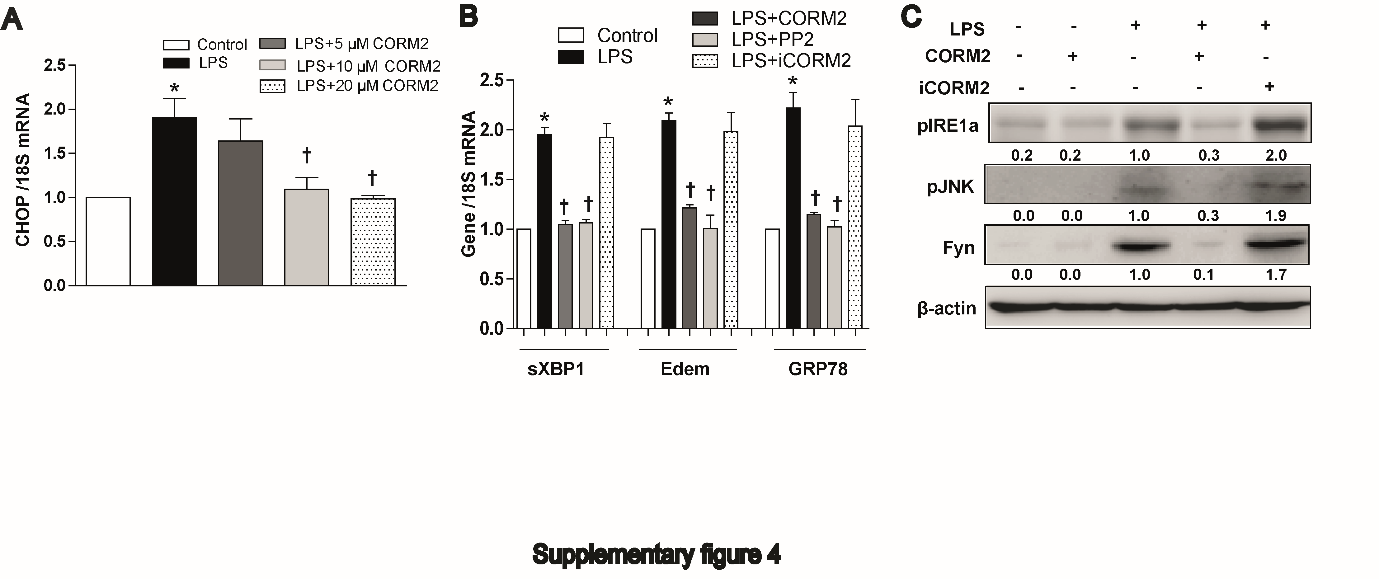


**Supplementary Fig. 4.** Effect of CORM2 on ER stress signaling in mProx cells. (A) Cells were pretreated with CORM2 in a concentration-dependent manner (0, 5, 10, and 20 µM) for 2 h and then stimulated with LPS (100 ng/ml) for 6 h. (A) CHOP mRNA. (B-C) Cells were pretreated with CORM2 (20 µM) or iCORM2 (20 µM, oxidized for 24 h at room temperature) or PP2 (10 µM) for 2 h and then stimulated with LPS (100 ng/ml) for 6 h or 18 h. (B) mRNAs of sXBP1, Edem, and GRP78 were measured at 6 h. (C) Proteins of pIRE1α, pJNK, and Fyn were measured at 18 h. All mRNAs were measured using real-time PCR and proteins were measured using western blotting analysis. All band intensities were measured using ImageJ software and the levels of the proteins were normalized to β-actin. Data are presented as mean ± SE, n=4. *p<0.05 vs. control, †p < 0.05 vs. LPS.


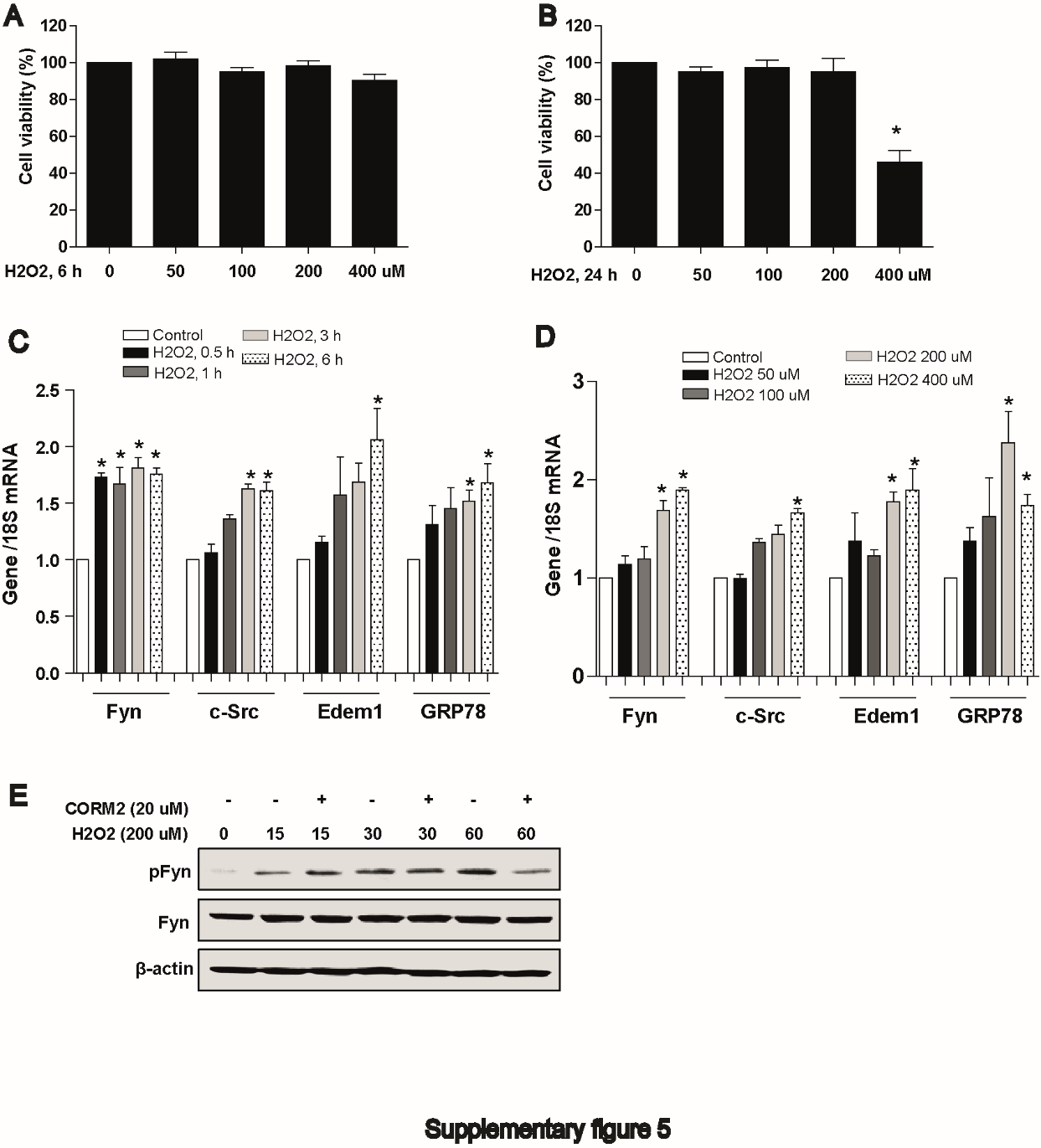


**Supplementary Fig. 5.** Effect of H_2_O_2_ on cell viability and ER stress responses in mProx cells. (A) Cells were stimulated with H_2_O_2_ in a dose-dependent manner (0, 50, 100, 200, and 400 µM) for 6 and 24 h and viability (%) was checked by MTT assay. (A) Cell viability (%) at 6 h and (B) cell viability (%) at 24 h. (C-D) Cells were stimulated with H_2_O_2_ in a time- and dose-dependent manner. (C) Fyn, c-Src, Edem, and GRP78 mRNA at different times (0, 0.5, 1, 3, and 6 h), and (D) Fyn, c-Src, Edem, and GRP78 mRNA at different doses (0, 50, 100, 200, and 400 µM) for 6 h. All mRNAs were measured using real-time PCR. (E) Cells were pretreated with CORM2 (20 µM) for 2 h and stimulated with H_2_O_2_ (200 µM) in a time dependent manner (0, 15, 30, and 60 min). Protein expression of pFyn and Fyn was measured using western blotting analysis. Representative protein bands are shown. Data are presented as mean ± SE, n=4. *p<0.05 vs. control.
